# Avatars in Mental Health: Psychotherapists’ Attitudes Towards Avatar Technology and Factors Influencing Adoption

**DOI:** 10.3390/ejihpe15120256

**Published:** 2025-12-13

**Authors:** Donatella Ciarmoli, Alessandro Gennaro, Francesca Lecce, Matteo Reho, Stefano Triberti

**Affiliations:** 1 LAHTI Laboratory for Advanced Human-Technology Interaction, Pegaso University, 20126 Milan, Italy; 2 Department of Psychology and Health Sciences, Pegaso University, 80143 Naples, Italy; alessandro.gennaro@unipegaso.it (A.G.); francesca.lecce@unipegaso.it (F.L.); stefano.triberti@unipegaso.it (S.T.); 3 Department of Biomedical, Metabolic and Neural Sciences, University of Modena and Reggio Emilia, 42121 Reggio Emilia, Italy; matteo.reho@unimore.it

**Keywords:** avatar, cybertherapy, psychotherapy, attitudes, acceptance, adoption, technology acceptance model

## Abstract

Research in “cybertherapy” has explored innovative ways to integrate new technologies as innovative tools in psychological treatment, such as virtual reality. Avatars, as digital representations of users within virtual environments, represent an interesting tool for psychotherapists: they could be used to assess aspects of patients’ self-representations (assessment), to promote behavioral change based on an alternative self-image (treatment), or to exercise therapists’ skills in diagnosis and assessment (formation). Yet, the use of avatars in psychotherapy is still not widespread. In the present study, 77 certified psychotherapists evaluated the three possible uses of avatars described above in terms of technology acceptance model (TAM) factors: perceived usefulness, perceived ease of use and intention-to-use. Partially confirming the TAM, the results show that perceived usefulness in particular is an effective predictor of intention to use avatars in psychotherapy for all three possible uses. Attitudes towards avatars as a psychotherapeutic tool were slightly influenced by mental health professionals’ methodological approach, with cognitive-behavioral psychotherapists showing more positive attitudes towards avatars as a training tool. On the other hand, previous experiences with other technologies (e.g., conducting therapy online or not) affected the perception of avatars’ ease of use as a treatment tool. The present study contributes to identifying factors that influence mental health professionals’ attitudes towards technological innovations in the psychotherapy profession, giving directions for future research in cybertherapy adoption.

## 1. Introduction

The way digital technology has woven itself into the fields of psychology and psychotherapy stands out as one of the biggest challenges and opportunities we have faced in recent decades. With the rise in interactive technologies, we have seen the emergence of new tools for assessment and clinical intervention that can really boost our diagnostic capabilities, engage patients more effectively, and enhance the overall impact of therapeutic change (Riva et al., 2021). Indeed, new technologies allow mental health professionals to design and employ new ways to generate meaningful experiences for patients that can be useful both for treatment and assessment. For example, virtual reality has recently been used for both diagnostic and therapeutic purposes.

On the diagnostic front, technologies such as virtual reality and digital behavioral tracking systems are increasingly being used to gather precise and ecologically valid data. For instance, VR-based scenarios can simulate social situations to assess phobic or avoidant responses, providing structured, repeatable, and immersive diagnostic environments (Geraets et al., 2022). Recent reviews show promising evidence of their effectiveness in improving the accuracy and sensitivity of clinical assessments (Valmaggia et al., 2016; Maples-Keller et al., 2017).

On the other hand, intervention tools have developed significantly. Recent meta-analyses confirm that exposure to virtual reality significantly reduces symptoms of anxiety or phobia compared to traditional interventions (Lim et al., 2023; Freitas et al., 2021). Similarly, social media are no longer seen only as an environment at risk for the onset of psychological and social problems; rather, they are considered as contexts in which benefits in mental well-being can be derived (Naslund et al., 2020). This overview indicates how clinicians have a digital toolbox (e.g., immersive environments for gradual exposure and social platforms for modulating daily interaction) with the ultimate goal of enhancing diagnostic accuracy and maximizing the effectiveness of therapeutic change (Wiederhold & Riva, 2022).

### 1.1. Avatars

In this panorama, avatars—digital representations of users within virtual environments—represent a useful opportunity to enrich psychological interventions through technologies. The use of an avatar concerns psychosis, where avatar therapy has shown promising effects in reducing the frequency and distress associated with auditory hallucinations (Craig et al., 2018). Other domains include anxiety disorders and phobias, where avatar- or VR-based exposure techniques have demonstrated efficacy in alleviating social anxiety and specific fears (Carl et al., 2019). In depression, digital programs integrating avatars as companions or mediators of cognitive-behavioral techniques have reported improvements in engagement and symptom reduction (Farrer et al., 2013). Research on autism spectrum disorders has also highlighted the potential of avatars to enhance social communication skills and emotional recognition in children and adolescents (Bellani et al., 2011). Across these conditions, emerging evidence indicates that avatar-based interventions can be effective and acceptable, although the robustness of findings varies depending on the clinical context and study design.

Avatars activate a process of projection, attribution, and prototyping of self-representations that can be potentially useful in contexts of psychological assessment, self-exploration, and therapeutic treatment (Waltemate et al., 2018; Fiedler et al., 2024). The use of avatars in psychology (especially in psychological research) has increased significantly in recent decades. In particular, Triberti and colleagues (Triberti et al., 2017, 2019) found that the process of avatar customization is an active identity-building activity, which can support the patient’s emotional involvement and facilitate the expression of personal elements that are difficult to verbalize. Numerous studies have demonstrated that avatars constitute reliable indicators of patients’ self-representation (Bessière et al., 2007), actual and idealized concepts of the selves, and personal and social identity (Zimmermann et al., 2023); of pathological or traumatic experience (Triberti et al., 2019; Riva et al., 2021; Vanderburg et al., 2023); and of the perception of specific social contexts and users’ intentions. For example, individuals may create different avatars based on different input by the experimenters or depending on the aim they are supposed to achieve within a virtual environment (e.g., working vs. having fun vs. dating) (Zimmermann et al., 2023; Vasalou & Joinson, 2009).

One of the most interesting theoretical approaches for understanding the utility of avatars in the psychological field is Self-Discrepancy Theory (Higgins, 1987). According to this theory, discrepancies between the real self, the ideal self, and the normative self can lead to negative emotions and psychological distress and act as predictors of psychopathology, e.g., addiction (Bessière et al., 2007). This framework has also been used to explore how individuals manage social anxiety in virtual settings and how body image concerns may influence avatar creation, especially in populations at risk for eating disorders or low self-esteem (Yee & Bailenson, 2007; De Vries et al., 2019). Self-discrepancy theory has been adapted to describe the identification and customization process in avatars (avatar-self discrepancy) (Dunn & Guadagno, 2012), and research based on its prescriptions confirms previous data based on self-description, self-drawing or other classical tools employed to study self-representation (Fong & Mar, 2015). These insights support the development of avatar-based interventions, where the avatar functions not only as a tool for self-expression but also as a potential entry point for therapeutic work on self-perception and identity integration.

### 1.2. Avatars in Clinical Psychology

On these bases, it is possible to prefigure uses of avatars in clinical psychology. Using avatars that visually represent these different versions of oneself can truly help raise awareness and stimulate therapeutic dialog. This allows patients to visualize and reflect on their own identity discrepancies (Fong & Mar, 2015; Bessière et al., 2007). This approach is particularly useful when verbal communication is not sufficient or is hindered, making the iconic and symbolic aspect of the avatar a truly valuable communicative resource. Avatars can serve as a “symbolic mediator” between therapist and patient, providing facilitated access to deep emotional content, interior experiences, and self-representations (Villani et al., 2016; Riva et al., 2021).

The Proteus effect (the tendency to think and act in ways that match the look of the avatar we inhabit) was first demonstrated when participants who were given taller or more attractive avatars behaved more confidently and disclosed more personal information than those with shorter or less attractive avatars (Yee & Bailenson, 2007). Since that seminal work, more than fifty experiments have replicated the phenomenon. A recent synthesis of those studies reports a small-to-medium average effect size (*r* ≈ 0.24) and notices that head-mounted displays make the Proteus effect even stronger than flat-screen setups (Ratan et al., 2020; Beyea et al., 2023). What matters for therapy is not only that the effect exists but that it can be directed toward concrete, real-world goals. Fox and Bailenson (2009) showed exactly that volunteers who watched a photorealistic avatar of themselves jogging were more likely to exercise during the following day. In other words, “trying on” a healthier or more confident self in VR can translate into measurable behavioral change offline. Transposed to a clinical setting, this insight opens a practical route for fostering change. A therapist might invite the client to sculpt an avatar that embodies the traits or emotional states they aspire to—a calmer posture, steadier eye contact, or a more resilient stance—and then rehearse difficult social scenarios or coping strategies inside a virtual environment. Because the client experiences those interactions in the first person, the session becomes a safe but vivid rehearsal that can lower anticipatory anxiety and strengthen self-efficacy. Early work on VR-enhanced treatments already highlights the added value of such embodied practices alongside traditional cognitive-behavioral or experiential techniques (Maples-Keller et al., 2017). In short, the Proteus effect supplies both a theoretical rationale and an empirical backbone for using customized avatars as active agents of therapeutic change: they are not merely diagnostic mirrors of the self, but experiential training grounds where new ways of feeling and functioning can be tried on, refined, and ultimately carried back into everyday life.

Despite this promising potential, the use of VR technologies still faces challenges rooted in clinicians’ attitudes and expectations, which have been extensively studied through the Technology Acceptance Model (TAM) (Davis, 1989). Even though digital technologies are now widespread, not every clinician seems to embrace or feel confident using these innovative tools in their practice (Topooco et al., 2017). To dig deeper into this issue, numerous studies have leaned on the TAM (Davis, 1989), which is a framework that looks at the factors that affect how new technologies are adopted. Originally, the TAM is based on the Theory of Planned Behavior, which posits that attitudes towards objects determine the intention to approach or avoid those objects, influencing actual behavior (Fishbein & Ajzen, 1975). The TAM revolves around two main predictors of the intention to use a technology: perceived usefulness (PU) and perceived ease of use (PEU). These attitudes have been widely acknowledged as key indicators of whether healthcare and psychology professionals are likely to use new technologies (Venkatesh & Davis, 2000; Alhur, 2022; Kung et al., 2024).

In the field of psychotherapy, the recent literature has begun to examine how interactive tools—such as apps, serious games, or virtual environments—can be effectively integrated into the therapeutic relationship (Maples-Keller et al., 2017). While the idea of using customizable avatars in psychotherapy is still a bit of a frontier, there have been some promising early studies showing their potential benefits. For instance, research has highlighted their effectiveness with oncology patients (Triberti et al., 2019), in treating eating disorders (Riva et al., 2021), and even in training for building empathetic relationships (Villani et al., 2016).

Avatars can now be thought of as a three-way bridge between assessment, intervention, and professional training. First, a growing stream of self-discrepancy work shows that the gap between a client’s actual attributes and the look or personality they give their avatar can reveal unmet needs and psychological distress; players in online worlds, for instance, consistently design characters closer to their ideal than to their real selves (Bessière et al., 2007). Mapping that “avatar-self discrepancy” has therefore become a quick, behaviorally anchored way to probe self-perception during intake or progress checks.

Second, avatars double as active agents of change. Experimental studies on the Proteus effect show that when people embody a taller, fitter, or more confident avatar, they almost immediately start to negotiate more assertively, disclose more, or exercise longer—changes that persist after the headset comes off (Yee & Bailenson, 2007; Fox & Bailenson, 2009). This makes avatar-based rehearsal a natural adjunct to cognitive-behavioral or exposure protocols: clients can “test-drive” desired behaviors in a safe, controllable space before trying them in vivo.

Third, avatar technology can be used to train the helpers themselves. Embodied-VR simulations that let trainees inhabit a patient’s perspective—or practice responding to a virtual client—have been shown to boost empathic accuracy and relational skills (Bertrand et al., 2018). For example, we can imagine designing evidence-based, prototypical patient avatars that psychotherapists in training may analyze to collect information relevant to diagnosis.

A key aspect to keep in mind when discussing the adoption of digital technologies in the clinical setting is the factors that influence how psychotherapists relate to these tools. Among the most significant is certainly the practitioner’s methodological orientation. Some studies indicate that more structured, behavior-focused approaches, such as cognitive-behavioral therapy (CBT), tend to welcome the use of technologies such as clinical apps, virtual reality or customizable avatars (Gega et al., 2013). This is because they align well with the principles of gradualism, monitoring and behavioral experimentation (Maples-Keller et al., 2017; Sebri et al., 2020). On the other hand, orientations that give more weight to the therapeutic relationship or symbolic interpretation might be more cautious, seeing the digital as something that could reduce or move away from the traditional clinical process (Jesser et al., 2022; Roesler, 2017; Scharff, 2013).

Another key factor is therapists’ prior experience with online psychotherapy. Those who have already had the opportunity to practice clinical interventions at a distance, even only occasionally, tend to consider digital tools as more useful and easier to use (Topooco et al., 2017). Direct experience helps to overcome initial fears related to technophobia or distrust of digital, promoting a more exploratory and hands-on attitude. Moreover, familiarity with digital environments can make it easier to understand the symbolic and communicative potential of innovative tools such as personalized avatars, especially when they are integrated into structured contexts and the therapeutic pathway.

This study is part of a larger research initiative that aims to delve into how psychologists and psychotherapists view the use of these avatars in three specific clinical settings: (1) as a projective tool during assessments, (2) as a supportive element during treatment, and (3) as a resource for teaching and training therapists. The theoretical model of reference is the TAM, supplemented by the assessment of digital skills using the Digital Competence Scale (DCS) (Scherer et al., 2019), to understand whether and how the level of familiarity with technology influences the acceptance of avatars. The present study was aimed at investigating Italian psychotherapists’ attitudes toward the adoption of avatar technology in clinical practice, guided by the Technology Acceptance Model (TAM). Specifically, the study intended to evaluate the influence of perceived usefulness (PU) and perceived ease of use (PEU) on the intention to use (IU) avatars for the three proposed applications: assessment, treatment, and training; to compare attitudes (PU, PEU, and IU) toward avatars across the three different clinical applications to identify potential differences in acceptance based on their use; to examine the influence of therapists’ methodological orientation (cognitive-behavioral vs. other approaches) on their acceptance of avatar technology across the different uses; finally, to investigate the impact of previous experience with online therapy on psychotherapists’ perceptions of the usefulness and ease of use of avatars.

By analyzing perceptions of usefulness and ease of use in each of these scenarios, the research aims to provide an empirical basis for understanding how and to what extent the use of avatars can be embraced and potentially integrated into clinical work, promoting a more interactive, symbolic, and identity-centered psychotherapy.

## 2. Materials and Methods

### 2.1. Participants

A total of 77 licensed mental health professionals were recruited, of whom 39 completed all study measures. The final sample comprised 30 women and 9 men, with a mean age of 38.16 years (*SD* = 9.70, range = 26–65 years). All participants were certified psychotherapists currently working in clinical settings throughout Italy.

Recruitment was carried out via professional mailing lists, university networks, and psychotherapy-focused online communities and social media groups. Inclusion criteria required (a) a valid license to practice psychotherapy in Italy and (b) at least one year of clinical experience: this was chosen in order to ensure that the psychotherapists in the sample had actual clinical experience beyond formal training, which in Italy takes around 3–4 years depending on the methodological approach. This criterion is also used often in the literature for psychotherapists involved in research (Brijwani & Desousa, 2023; Pischel et al., 2025; Mander et al., 2013). Clinicians who were not actively working in clinical settings were excluded. In general, the sampling strategy was more effective when it involved specialized communities accessed by some of the authors, who are practicing psychotherapists. As said above, 39 participants responded to all study measures. Specifically, part of the sample chose not to respond to questions about psychotherapy methodological approach and online therapy experience, possibly because they did not feel represented by available response options.

44 participants completed the Digital Competence Scale (DCS) (see Section 2.2) and revealed a notably high affinity with digital technology across the assessed areas (information and data literacy, communication and collaboration, digital content creation, safety, and problem solving): mean = 6.07, *SD* = 0.77, range 3.7–7, with 7 being the highest possible score.

### 2.2. Instruments

#### 2.2.1. Technology Acceptance Model (TAM) Questionnaire

To gauge clinicians’ acceptance of customizable avatars in psychotherapy, we adapted the Technology Acceptance Model (TAM) questionnaire (Davis, 1989; Venkatesh & Davis, 2000). A prototypical item from the TAM is, for example, ‘Using [this system] improves my performance in [the task].’ This illustrates how TAM items are generally formulated and clarifies why the scale is inherently designed to be adaptable to different contexts. The instrument contained nine 7-point Likert items (1 = extremely unlikely, 7 = extremely likely). The full list of adapted items is reported in Appendix A (see Table A1). Items 1–4 indexed perceived usefulness (PU), items 5–8 perceived ease of use (PEU), and item 9 behavioral intention to use. In the present study, mean scores were calculated separately for PU and PEU. The original instrument showed excellent internal consistency (PU α = 0.98, PEU α = 0.94; Davis, 1989), with later studies confirming reliability coefficients above 0.80 (Venkatesh & Davis, 2000). In the present study, both PU and PEU scales showed internal consistency of 0.96.

#### 2.2.2. Clinical Scenario Vignettes

Three textual vignettes (equal length) described potential applications of customizable avatars in psychotherapy (see Table A2: “Clinical scenario vignettes” in Appendix A): (a) Assessment—Avatars used as projective tools to explore a client’s traits, emotions, and identity facets; (b) Therapeutic intervention—Avatars employed within treatment to facilitate emotional expression, self-discrepancy work, and symbolic elaboration; and (c) Training—Avatars simulating clinical interactions or supporting the acquisition of therapeutic techniques.

The vignette method has been adopted in light of its eliciting attitudes and decision-making through standardized hypothetical scenarios, particularly when investigating emerging technologies that are not yet accessible for direct experience (Finch, 1987; Schoenberg & Ravdal, 2000).

Each vignette was followed by the nine TAM items, yielding attitude ratings for the three distinct clinical contexts. Additional questions collected demographic data, therapeutic orientation (open-ended), and previous experience with online therapy (yes/no).

#### 2.2.3. Digital Competence Scale (DCS)

To capture clinicians’ self-rated digital skills, we used the Digital Competence Scale (DCS) (Scherer et al., 2019). The DCS is a brief 12-item self-assessment instrument grounded in the European DigComp framework. Items are rated on a 7-point Likert scale (1 = strongly disagree, 7 = strongly agree), covering five competence areas: information and data literacy, communication and collaboration, digital content creation, safety, and problem solving. The original validation study showed excellent internal consistency (α = 0.93) and strong convergent validity with performance-based ICT tests. In the present study, a mean total score was computed, with higher values indicating greater perceived digital competence (see Section 2.1).

### 2.3. Procedures

Eligible clinicians received a unique Qualtrics link distributed via e-mail or private messages. After opening the link on a desktop or mobile device (the survey is fully responsive), participants were first presented with an electronic informed consent page and a brief definition of “avatar” (to distinguish user-controlled avatars from computer-controlled embodied agents). For transparency, the full wording of this definition is reported in both the original Italian and its English translation in Appendix A (see Table A3).

The three vignettes were presented in randomized order to control for sequence effects. For each vignette, respondents completed the nine TAM items before advancing to the next scenario. After rating all scenarios, they filled out the DCS questionnaire and items on demographic and professional information. Specifically, psychotherapists were asked to report their methodological approach (i.e., whether they employed an approach more associated with cognitive-behavioral, psychodynamic, humanistic, systemic relational, or other) and whether they offered online consultation or not to their patients. Upon finishing, data were stored securely on the Qualtrics (2025) platform and later exported to statistical-analysis software, IBM Corp. (2025) SPSS v.30.

### 2.4. Ethical Considerations

The study received approval from the Ethical Committee of Pegaso University (protocol PROT./E 003392). All procedures conformed to the Declaration of Helsinki. Participation was voluntary and anonymous; respondents could withdraw at any point without penalty. Data were encrypted and retained on password-protected servers accessible only to the research team.

### 2.5. Data Analysis

Data analysis was conducted with the software IBM Corp. (2025) SPSS v.30. First, a factor analysis was performed in order to ensure that the items used to investigate perceived utility (PU) and perceived ease of use (PEU) would show adequate internal consistency over two different factors. A principal component analysis with varimax rotation was conducted on all 24 items (4 PU items and 4 PEU items employed three times to assess attitudes towards avatars for assessment, treatment, and training). In order to assess hypotheses, three regression analyses were performed based on the technology acceptance model (TAM); specifically, the subscales PU (perceived usefulness) and PEU (perceived ease of use) were computed and used as predictors, along with DCS, of intention to use the technology. The three regression models regarded the three possible uses of avatars in mental health (assessment, treatment and training).

Second, we compared the PU attitudes (perceived usefulness) and PEU (perceived ease of use), as well as intention-to-use, across the three uses of avatars within the mental health profession by means of repeated measures ANOVA in order to assess differences in attitudes based on the possible uses of avatars in psychotherapy.

Third, mixed design ANOVAs were conducted in order to assess the influence of psychotherapists’ methodological orientation and experience vs. no experience with online therapy on attitudes towards avatars in psychotherapy. Regarding the first variable, we found that the distribution of therapeutic orientations in the sample only allowed for a comparison between cognitive-behavioral therapists and all other orientations combined, as the number of participants in each of the other approaches was too small for separate analyses.

The significance threshold was set at α = 0.05 for all statistical tests.

### 2.6. Factor Analysis

The factor analysis on items used to investigate Perceived utility (PU) and Perceived ease of use demonstrated good internal consistency of the items, loading on two factors consistent with hypotheses (see Table 1). The explained variance of the factors was 47.1% for PU and 25.9% for PEU.

## 3. Results

### 3.1. Regression Analyses

As shown in Table 2, all the models were significant, but only PU emerged as a significant predictor of intention to use avatars in mental health profession activities. Notably, all explained variance values were above 70%, highlighting the importance of PU in influencing the use of avatars in psychotherapy practice.

### 3.2. Repeated-Measures ANOVAs

Concerning the within-subjects comparisons between avatar uses (Table 3 features mean values), the first comparison between PU attitudes was not significant, indicating that no statistically significant differences emerged in perceived utility of avatars across the three possible uses: *F*(2, 88) = 2.033, *p* = 0.137. Also, the comparison between intentions to use the avatar technology was not statistically significant: *F*(2, 88) = 0.407, *p* = 0.667. The means reported in Table 2 show an average medium-to-low interest towards using avatars in psychotherapy in our sample.

Diversely, the comparison regarding PEU (perceived ease of use) was significant: *F*(2, 88) = 3.668, *p* = 0.029, η^2^ = 0.077, highlighting that avatars were considered less easy to use in treatment compared with the other uses. Specifically, post hoc pairwise comparisons with Bonferroni correction indicated that Treatment differed significantly from Training (*p* = 0.030), while the difference between Treatment and Assessment was not significant (*p* = 0.404) and the difference between Assessment and Training was not significant (*p* = 0.872).

### 3.3. Mixed-Design ANOVAs: Therapeutic Orientation and Online Therapy Experience

Third, to verify whether attitudes toward avatars differed between cognitive-behavioral (CBT) therapists and therapists from other approaches, three mixed-design 2 × 3 ANOVAs were conducted on (a) intention to use, (b) perceived usefulness (PU), and (c) perceived ease of use (PEU) (see Table 4 and Table 5).

With regard to perceived usefulness (PU), the interaction between Therapist Orientation × avatar possible use (training, treatment, and assessment) showed a trend toward significance (*p* = 0.053), but did not reach the predefined threshold of 0.05—*F*(2, 76) = 3.055, *p* = 0.053, η^2^ = 0.074—suggesting that cognitive therapists consider avatars more useful in training (*M* = 3.99, *SD* = 1.53) compared to their colleagues from other orientations (*M* = 3.33, *SD* = 1.66). The main effect of therapeutic orientation was negligible (*F*(1, 38) = 0.65, *p* = 0.427).

Regarding oerceived ease of use (PEU), the interaction was significant—*F*(2, 76) = 3.626, *p* = 0.031, η^2^ = 0.089—indicating that non-cognitive therapists perceive avatars as less intuitive, especially in the treatment context (*M* = 4.07 ± 1.76 vs. 4.67 ± 1.65 for cognitive therapists). The main effect of therapist orientation remained non-significant (*F*(1, 38) = 1.46, *p* = 0.235).

Finally, regarding intention to use, no differences emerged: neither the interaction between Therapist Orientation × Avatar possible use (*F*(2, 76) = 0.20, *p* = 0.819, η^2^ = 0.005) nor the main effect (*F*(1, 38) = 0.17, *p* = 0.686) reached statistical significance; the average interest in adopting avatars remained consistently medium-low across both groups.

In summary, theoretical orientation has a limited impact: CBT therapists show a slightly higher tendency to recognize the usefulness and ease of use of avatars (particularly for training purposes), but this does not translate into an increased intention to adopt them.

To investigate the influence of online clinical experience on attitudes toward the use of avatars, three mixed-design 2 × 3 ANOVAs were conducted (between-subjects variable: whether or not clinicians conduct online psychotherapy; within-subjects variable: possible avatar use; dependent variables: one for each TAM variable).

Regarding PU, the interaction between possible avatar uses and clinicians conducting online therapy or not was not significant (*F*(2, 74) = 0.34, *p* = 0.966, η^2^ = 0.040). Concerning PEU, the interaction effect was significant (*F*(2, 74) = 3.626, *p* = 0.031 *, η^2^ = 0.089) (see Table 5 for descriptives). Psychotherapists who reported having online therapy experience considered avatars less easy to use for treatment purposes.

Finally, with respect to IU, the interaction was not significant (*F*(2, 74) = 0.47, *p* = 0.954, η^2^ = 0.001). The intention to adopt avatar technology remained unchanged between the two groups.

## 4. Discussion

The results of this study offer insights into the dynamics influencing the acceptance of digital avatars among mental health professionals as a specific but multi-functional and innovative tool for clinical interventions. According to the TAM, perceived usefulness (PU) proved to be a significant predictor of intention-to-use in all three contexts examined (assessment, treatment and training), whereas perceived ease of use (PEU) had no significant impact. This suggests that, for clinicians, the perceived clinical usefulness of avatars is more important than ease of use when it comes to deciding whether to integrate them into their professional practice. This is consistent with existing research employing TAM to evaluate different technologies, as perceived ease of use is sometimes regarded as the most controversial predictor of intention to use a technology and does not always appear relevant (Tan et al., 2012; Chua & Yu, 2024). It should be noted that no statistically significant differences emerged in the perceived usefulness of avatars across the three application domains (evaluation, treatment, and training). This result should not be interpreted as evidence of equal usefulness but rather as the absence of significant differences in this sample. For example, for expert users of sophisticated/professional technology, perceived ease of use can be irrelevant or even counterproductive, as they may feel undermined in their expertise by a “user-friendly” interface when they are used to learning and using sophisticated tools (Triberti & Brivio, 2017). At the same time, when people are requested to express their opinion towards a mere description of a new technology, ease of use can be more difficult to represent than general utility.

Despite the growing scientific interest in avatars as tools for self-exploration and therapeutic mediation (Riva et al., 2021; Triberti et al., 2019; Rehm et al., 2016; Jang et al., 2025), the intention to use expressed by participants remained moderate overall. This suggests that there are still cultural, technical or educational barriers to overcome. The use of avatars is not perceived as immediately intuitive, especially in the context of treatment, where the relationship between therapist and patient is central and not always easily digitized.

A deeper analysis revealed that therapeutic orientation plays a slight role in the acceptance of avatars. Therapists with a cognitive-behavioral approach may consider avatars more useful, especially in the context of training, than colleagues from other orientations. This could be due to the structured and goal-oriented nature of CBT, which lends itself better to the integration of digital tools. Previous research has shown that CBT is more likely to adopt innovative technologies due to its emphasis on standardized and measurable protocols (Maples-Keller et al., 2017). For example, cognitive-behavioral therapists were more positive towards the utilization of AI in therapy than systemic and psychoanalytic colleagues (Sebri et al., 2020). As noted in the data analysis section, it was not possible due to the methodological approaches available within the sample to compare the attitudes of other approaches more specifically. Although the observed differences may be related to participants’ therapeutic orientation, other sample characteristics (e.g., age, professional experience, or technological affinity) could also have influenced the results. Future studies should control for these factors more systematically.

On the contrary, prior experience in conducting online therapy had an influence on perceived ease of use of avatars, but in a different direction than what was expected. Psychotherapists with experience in online therapy considered avatars less easy to use for treatment purposes. This result contrasts with some previous research that suggested a greater openness towards digital tools by clinicians with online therapy experience (Topooco et al., 2017). However, it is possible that experience with online psychotherapy does not automatically translate into a greater inclination to adopt more immersive technologies such as avatars, which can possibly be employed independently of therapy taking place over online sessions. Psychotherapists with online therapy experience may be more sensitive to fine-tuned aspects of their online sessions: they may feel that using avatars to influence behavior change, for example, within virtual simulations, may be more difficult to manage online. Indeed, as Sayers (2021) reports when reflecting on colleagues’ and her own professional experience, online psychotherapy may be affected by technical issues, interruptions, and difficulties in communication that could affect the therapeutic alliance: it is possible that the online psychotherapists in the sample reflected on similar issues while assessing the ease of use of avatars for treatment purposes.

### Limitations

The present study has some limitations that must be highlighted. First, the evaluation of avatar technology was based solely on textual vignettes rather than hands-on experience or visual demonstrations. Consequently, therapists’ perceptions were formed in response to a hypothetical scenario and may not accurately reflect their attitudes toward using actual avatar systems in a real clinical setting. The lack of direct interaction with the technology may have particularly influenced ratings of perceived ease of use (PEU), as participants could not experience the interface’s intuitiveness firsthand, potentially explaining its non-significant role in predicting usage intention. Second, the small sample size, although adequate for the statistical analyses conducted, limits the generalizability of the findings and the power to detect smaller effects. Third, the sample was composed of self-selected volunteers, which may indicate a pre-existing interest or openness toward technology, potentially introducing a selection bias. Therefore, the results might represent a more positive outlook on avatar technology than that held by the broader population of psychotherapists in Italy. Another limitation could be ascribed to sampling. Part of the sample chose not to respond to the question about methodological orientation. We hypothesize that they may not feel represented by the provided options (e.g., “hybrid” methodological approaches). Future research on psychotherapists’ attitudes may use open questions to allow psychotherapists to describe their approach, should it not coincide with the most widespread approaches.

Finally, the study’s cross-sectional design provides a snapshot of attitudes at a single point in time. Attitudes toward novel technologies are dynamic and can evolve with increased exposure, training, and demonstrable evidence of their clinical efficacy.

Future research should adopt longitudinal designs to track changes in therapists’ perceptions as their exposure to avatar systems grows. Larger and more diverse samples will be essential to capture the heterogeneity of professional contexts, cultural backgrounds and different clinical orientations. Experimental studies offering direct interaction with avatar technologies could clarify the role of usability and experiential learning in shaping acceptance. Moreover, comparative investigations with other forms of digital interventions, as well as clinical outcome studies, could shed light on the specific added value of avatars in psychotherapy. Such directions would not only validate and extend the present findings but also contribute to building an evidence base that informs training programs, technology design, and clinical implementation.

## 5. Conclusions

This study represented the first empirical investigation of Italian psychotherapists’ attitudes towards the use of customizable avatars in clinical settings. The results clearly show that perceived usefulness (PU) is the main predictor of intention to use, while perceived ease of use (PEU) has a negligible influence. This confirms what is suggested by the TAM (Davis, 1989; Venkatesh & Davis, 2000), according to which perceived usefulness is crucial for the adoption of new technologies, even in complex professional fields such as mental health.

Besides adoption, this study is interesting to achieve a glimpse of factors that could influence psychotherapists’ attitudes towards innovative tools that could be implemented in the profession. An interesting aspect that emerged is that the theoretical orientation of the therapist influences their perceptions: consistently with the literature, it is possible that clinicians’ methodological approach influences their perception of tools and technologies. Cognitive-behavioral psychotherapists tend to recognize the usefulness and ease of use of avatars more, especially for clinical training. This is in line with the literature that considers CBT approaches more open to the integration of digital tools due to their structured and pragmatic nature (Maples-Keller et al., 2017; Sebri et al., 2020). As a future research direction, studies may explore more methodologically diverse samples of psychotherapists to highlight opinions specific to other approaches.

Furthermore, the previous experience with online psychotherapy could also influence mental health professionals’ attitudes towards new technologies, but not necessarily in a positive way. Instead of being simply more open towards technologies in therapy, psychotherapists with technological experience may assess the feasibility of more sophisticated scenarios (e.g., avatars for treatment would probably be based on interactive virtual simulations) more critically. It should be noted that the current study did not collect information about the perceived quality or satisfaction of psychotherapists with online consultation, which could be important to understand their opinion towards other technological innovations. Future research could take this into consideration in order to better explain mental health professionals’ attitudes.

In the light of these findings, it is clear that it is crucial to design specific training interventions that not only improve practitioners’ digital skills (Scherer et al., 2019) but that are also able to enhance the symbolic and transformative potential of avatars in the clinical context. As indicated by Fox and Bailenson (2009), when a patient has the opportunity to “experience” an enhanced version of himself or herself through an avatar, this can lead to lasting behavioral changes in real life as well. Avatars, therefore, assume a dual role: that of a diagnostic mirror and a tool for experiential change (Yee & Bailenson, 2007; Ratan et al., 2020).

In conclusion, this work represents a valuable starting point for exploring how to integrate avatars into psychotherapy, suggesting that the adoption of these tools is not only a technical issue but also involves identity, epistemological and relational aspects that deserve further investigation. Implementation projects that would like to promote the usage of new technologies in a sensitive field such as mental health should take into account therapists’ methodological approaches, pre-existing attitudes and experience, possibly to guide the design of the technologies based on the actual needs and practices within the profession. Future research could broaden the sample, examine concrete application contexts and also investigate the ethical and relational dimensions of these technologies in the therapeutic setting.

## Figures and Tables

**Table 1:** Factor loadings from principal component analysis with varimax rotation.

Variables	Factor 1 (PU)	Factor 2 (PEU)
Assessment PU1: Using this technology would improve my performance in doing my job	**0.896**	0.168
Assessment PU2: Using this technology in my job would increase my productivity	**0.941**	0.102
Assessment PU3: Using the technology would increase my effectiveness in my job	**0.942**	0.100
Assessment PU4: I think this technology would be useful in my work	**0.926**	0.129
Assessment PEU1: Learning how to use the technology would be easy for me	0.068	**0.943**
Assessment PEU2: I would find it easy to make the technology do what I want it to do	0.195	**0.899**
Assessment PEU3: It would be easy for me to become proficient in using this technology	0.165	**0.928**
Assessment PEU4: I think the described technology would be easy to use	0.079	**0.939**
Treatment PU1	**0.928**	0.176
Treatment PU2	**0.944**	0.113
Treatment PU3	**0.878**	0.273
Treatment PU4	**0.866**	0.304
Treatment PEU1	0.139	**0.890**
Treatment PEU2	0.335	**0.767**
Treatment PEU3	0.178	**0.916**
Treatment PEU4	0.200	**0.932**
Training PU1	**0.895**	0.172
Training PU2	**0.936**	0.056
Training PU3	**0.963**	0.102
Training PU4	**0.892**	0.295
Training PEU1	0.086	**0.882**
Training PEU2	0.205	**0.858**
Training PEU3	0.120	**0.926**
Training PEU4	0.155	**0.852**

**Table 2:** Regression analyses on the three possible uses of avatars in mental health according to the Technology Acceptance Model (TAM).

Variables	β	t	*p*
Avatar for Assessment *F* = 79.59, *p* < 0.001, R^2^ = 0.71			
PU (Perceived Utility)	0.836	11.921	<0.001
PEU (Perceived Ease of Use)	0.054	0.773	0.442
Avatars for Treatment *F* = 71.61, *p* < 0.001, R^2^ = 0.71			
PU (Perceived Utility)	0.856	10.763	<0.001
PEU (Perceived Ease of Use)	−0.022	−0.275	0.784
Avatars for Training *F* = 85.19, *p* < 0.001, R^2^ = 0.75			
PU (Perceived Utility)	0.867	12.315	<0.001
PEU (Perceived Ease of Use)	0.008	0.107	0.915
Outcome variable: Intention to use			

**Table 3:** Means and standard deviations from the comparisons between attitudes and intention-to-use towards avatars across the three possible uses in psychotherapy.

Variables	Assessment (Mean, *SD*)	Treatment (Mean, *SD*)	Training (Mean, *SD*)
Perceived Utility *N* = 45, *F*(2, 88) = 2.033, *p* = 0.137	3.5, 1.5	3.4, 1.5	3.7, 1.6
Perceived Ease of use * N = 45, *F*(2, 88) = 3.668, *p* = 0.029	4.6, 1.5	4.4, 1.6	4.7, 1.4
Intention-to-Use *N* = 45, *F*(2, 88) = 0.407, *p* = 0.667	3.2, 1.7	3.1, 1.7	3.2, 1.7

* = significant difference.

**Table 4:** Means and standard deviations (*M* ± *SD*) for Perceived Usefulness (PU), Perceived Ease of Use (PEU), and Intention to Use, divided by therapeutic orientation (cognitive-behavioral vs. non-cognitive) and avatar application context (assessment, treatment, training).

Variables	Assessment (Mean, *SD*)	Treatment (Mean, *SD*)	Training (Mean, *SD*)
Perceived usefulness *N* = 39, *F*(2, 76) = 3.055, *p* = 0.053, η^2^ = 0.074			
Cognitive Orientation	3.36, 1.42	3.63, 1.48	3.99, 1.53
Other Orientation	3.45, 1.65	3.10, 1.53	3.33, 1.66
Perceived ease of use *N* = 39, *F*(2, 76) = 3.626, *p* = 0.031, η^2^ = 0.089			
Cognitive Orientation	4.88, 1.49	4.67, 1.65	5.13, 1.46
Other Orientation	4.45, 1.78	4.07, 1.76	4.40, 1.55
Intention to use *N* = 39, *F*(2, 76) = 0.20, *p* = 0.819, η^2^ = 0.005			
Cognitive Orientation	3.21, 1.62	3.05, 1.72	3.21, 1.72
Other Orientation	3.14, 1.82	2.90, 1.70	2.86, 1.59

**Table 5:** Means and standard deviations (*M* ± *SD*) of Perceived Usefulness (PU), Perceived Ease of Use (PEU), and Intention to Use, divided based on whether clinicians had experience with online psychotherapy (yes vs. no) and avatar use (assessment, treatment, training).

Variables	Assessment (Mean, *SD*)	Treatment (Mean, *SD*)	Training (Mean, *SD*)
Perceived usefulness *N* = 39, *F*(2, 74) = 0.34, *p* = 0.966, η^2^ = 0.040			
Online experience	2.8, 1.3	2.7, 1.4	3.1, 1.5
No online experience	3.9, 1.5	3.8, 1.4	4.1, 1.5
Perceived Ease of use * N = 39, *F*(2, 74) = 3.626, *p* = 0.031, η^2^ = 0.089			
Online experience	4.6, 1.7	4.0, 1.9	4.8, 1.5
No online experience	4.7, 1.6	4.6, 1.4	4.7, 1.5
Intention-to-Use *N* = 39, *F*(2, 76) = 0.47, *p* = 0.954, η^2^ = 0.001			
Online experience	2.7, 1.6	2.6, 1.6	2.7, 1.8
No online experience	3.4, 1.6	3.2, 1.7	3.2, 1.5

* = significant difference.

## Data Availability

The raw data supporting the conclusions of this article will be made available by the authors on request.

## Abbreviations

The following abbreviations are used in this manuscript:

TAM	Technology Acceptance Model
PU	Perceived Usefulness
PEU	Perceived Ease of Use
CBT	Cognitive-Behavioral Therapy

## Appendix A

**Table A1:** Adapted Technology Acceptance Model (TAM) items.

Using this technology would improve my performance in doing my job. Using this technology in my job would increase my productivity. Using the technology would increase my effectiveness in my job. I think this technology would be useful in my work. Learning how to use the technology would be easy for me. I would find it easy to make the technology do what I want it to do. It would be easy for me to become proficient in using this technology. I think the described technology would be easy to use. I would like to use this technology regularly in my work.

**Table A2:** Clinical-scenario vignettes.

Potential Applications of Customizable Avatars in Psychotherapy	Original Vignettes in Italian	Vignettes Translated into English
Assessment	La ricerca mostra che le persone creano avatar per rappresentare aspetti della propria identità e/o in funzione delle proprie intenzioni nei confronti dell’ambiente virtuale. È dimostrato che caratteristiche degli avatar, così come gli atteggiamenti che le persone riportano verso gli avatar che loro stesse hanno creato, si associano significativamente a stati clinicamente significativi, come ansia e depressione. Si ritiene possibile utilizzare questa tecnologia come strumento aggiuntivo di assessment psicologico, per aiutare i pazienti a far emergere aspetti importanti della rappresentazione di sè. Uno psicoterapeuta può analizzare il risultato finale della creazione di uno o più avatar (in modo più o meno proiettivo o quantitativo), e/o accompagnare il paziente nel processo di creazione dell’avatar come stimolo per far emergere le sue rappresentazioni. Cosa pensi di questa tecnologia?	Research shows that people create avatars to represent aspects of their identity and/or according to their intentions within the virtual environment. It has been demonstrated that avatar characteristics, as well as the attitudes people report toward the avatars they have created, are significantly associated with clinically relevant states such as anxiety and depression. This technology is considered potentially useful as an additional tool for psychological assessment, helping patients to bring out important aspects of their self-representation. A psychotherapist can analyze the final outcome of creating one or more avatars (in a more or less projective or quantitative way), and/or accompany the patient through the process of avatar creation as a stimulus to elicit their representations. What do you think about this technology?
Training/formation	La formazione in psicoterapia beneficia sempre più di strumenti innovativi, tra cui simulazioni e ambienti virtuali immersivi, che consentono ai terapeuti in formazione di esercitare le proprie abilità diagnostiche e relazionali in modo sicuro, standardizzato e ripetibile. In questo contesto, l’utilizzo di avatar personalizzabili con caratteristiche psicologiche predefinite può rappresentare uno strumento particolarmente utile. Ad esempio, un formatore potrebbe creare una serie di avatar che incarnano varie tipologie di pazienti virtuali, ciascuno con tratti, sintomi e pattern relazionali specifici (ad esempio: un avatar con evidenti sintomi ansioso-depressivi…). I terapeuti in formazione avrebbero così la possibilità di “incontrare” e sperimentarsi nell’interazione con diverse tipologie di pazienti. Attraverso questo processo, i futuri terapeuti possono imparare a formulare ipotesi diagnostiche e sviluppare piani di intervento. In sintesi, gli avatar diventano una sorta di “paziente virtuale” utile come palestra formativa. Cosa pensi di questa tecnologia?	Psychotherapy training increasingly benefits from innovative tools, including simulations and immersive virtual environments, which allow trainee therapists to practice their diagnostic and relational skills in a safe, standardized, and repeatable way. In this context, the use of customizable avatars with predefined psychological characteristics can represent a particularly useful tool. For example, a trainer could create a series of avatars that embody different types of virtual patients, each with specific traits, symptoms, and relational patterns (e.g., an avatar with evident anxiety-depressive symptoms…). In this way, trainee therapists would have the opportunity to ‘meet’ and practice interacting with various types of patients. Through this process, future therapists can learn to formulate diagnostic hypotheses and develop intervention plans. In short, avatars become a kind of ‘virtual patient’ that serves as a valuable training ground. What do you think about this technology?
Therapeutic intervention	La ricerca mostra come le caratteristiche dell’avatar possano influenzare la percezione di sé e il comportamento dell’individuo che lo utilizza: per esempio, l’utilizzo di avatar alti rende gli utenti più dominanti e aggressivi, così come l’utilizzo di avatar attraenti rende più aperti e socievoli. Studi mostrano anche che i cambiamenti appresi nell’ambiente virtuale attraverso l’uso di avatar si estendono nell’ambiente reale. In un contesto terapeutico, questa tecnologia può essere sfruttata per promuovere il cambiamento psicologico desiderato. Ad esempio un paziente potrebbe utilizzare avatar forniti dl terapeuta per raggiungere obbiettivi clinici (ad esempio: maggiore sicurezza in sé, minore ansia sociale, maggiore assertività o resilienza). Tale esperienza virtuale garantita dalla tecnologia potrebbe promuovere l’interiorizzazione di nuovi schemi comportamentali, emozionali o di coping. Cosa pensi di questa tecnologia?	Research shows how avatar characteristics can influence self-perception and the behavior of the individual using them: for example, using tall avatars makes users more dominant and aggressive, while using attractive avatars makes them more open and sociable. Studies also show that changes learned in the virtual environment through the use of avatars extend into the real environment. In a therapeutic context, this technology can be leveraged to promote the desired psychological change. For instance, a patient could use avatars provided by the therapist to achieve clinical goals (e.g., greater self-confidence, reduced social anxiety, increased assertiveness, or resilience). Such a virtual experience enabled by technology could foster the internalization of new behavioral, emotional, or coping patterns. What do you think about this technology?

**Table A3:** Brief definition of “avatar” provided to participants.

Italian Version	English Version
Un avatar può essere definito come una rappresentazione digitale di una persona, creata per interagire in ambienti virtuali o digitali. Gli avatar non si limitano a essere una replica fisica di chi li utilizza, ma spesso si configurano come una forma di espressione della propria identità, delle proprie aspirazioni o persino delle proprie fantasie. L’avatar può riflettere caratteristiche personali, preferenze o aspetti immaginari che l’individuo desidera esplorare. L’avatar, in quanto rappresentazione digitale e personalizzabile del sé, introduce nuove opportunità che vanno oltre i limiti delle tecniche tradizionali, permettendo una personalizzazione complessa, offrendo una gamma più ampia di possibilità espressive rispetto a un semplice disegno, infatti, gli individui possono scegliere dettagli estetici, comportamentali e simbolici che riflettono in modo più autentico il loro vissuto.	An avatar can be defined as a digital representation of a person, created to interact in virtual or digital environments. Avatars are not limited to being a physical replica of their user; rather, they often serve as a form of expression of one’s identity, aspirations, or even fantasies. An avatar can reflect personal characteristics, preferences, or imaginary aspects that the individual wishes to explore. As a customizable digital representation of the self, the avatar introduces new opportunities that go beyond the limits of traditional techniques, enabling complex personalization and offering a broader range of expressive possibilities compared to a simple drawing. Indeed, individuals can choose aesthetic, behavioral, and symbolic details that more authentically reflect their lived experience.
